# Stem Cell-Based Strategies for Fibrotic and Neurogenic Bladder Disorders: Current Evidence, Translational Challenges, and Future Directions

**DOI:** 10.3390/cimb48070658

**Published:** 2026-06-26

**Authors:** Jae Heon Kim, Miho Song, Yun Seob Song

**Affiliations:** Department of Urology, Soonchunhyang University School of Medicine, Seoul 140-743, Republic of Korea; piacekjh@hanmail.net (J.H.K.); miho@schmc.ac.kr (M.S.)

**Keywords:** bladder fibrosis, mesenchymal stem cells, drug delivery, hepatocyte growth factor, neurogenic bladder, spinal cord injury

## Abstract

Progressive bladder fibrosis and impaired detrusor function represent converging pathological endpoints across diverse bladder disorders, including bladder outlet obstruction (BOO) associated with benign prostatic hyperplasia, spinal cord injury (SCI)-induced neurogenic bladder, radiation cystitis, and interstitial cystitis/bladder pain syndrome. Conventional therapies primarily manage symptoms and rarely reverse established fibrosis or restore durable bladder homeostasis. Mesenchymal stem/stromal cells (MSCs) have attracted considerable interest as therapeutic agents owing to their antifibrotic, immunomodulatory, angiogenic, and trophic paracrine activities. This review synthesises six key studies from our group and places them within the broader international literature on bladder regenerative medicine: (i) feasibility of superparamagnetic iron oxide (SPIO)-based molecular MRI tracking of transplanted human MSCs (hMSCs) in the bladder; (ii) SPIO-hMSC therapy for BOO-associated fibrosis with concurrent MRI monitoring; (iii) hepatocyte growth factor (HGF)-overexpressing engineered hMSC (B10.HGF) therapy in BOO; (iv) hMSC transplantation into the SCI-injured bladder wall monitored by MRI; (v) systematic review and meta-analysis of stem cell therapy effects on urodynamic outcomes in SCI models; and (vi) HGF-overexpressing hMSC therapy for BOO-induced underactive bladder. These six key studies are contextualised within the broader literature addressing cell sources, biomaterial-assisted delivery platforms, mechanistic pathways, emerging clinical evidence, and the evolving regulatory landscape for cell-based advanced therapy medicinal products. Key translational challenges include product standardisation, long-term durability, and mechanism-linked potency assay development.

## 1. Introduction

Bladder diseases culminating in pathological tissue remodelling—characterised by hypertrophy, chronic inflamma-tion, extracellular matrix accumulation, and fibrosis—impose substantial long-term morbidity by irreversibly com-promising compliance, safe storage, and effective emptying, thereby increasing risks of urinary tract infection, urinary retention, upper tract deterioration, pain syndromes, and diminished quality of life [1,2].

This review is structured as a narrative translational synthesis integrating six key studies from our group [3,4,5,6,7,8] and placing them within the broader context of contemporary stem cell research in bladder regeneration [9,10,11].

Progressive bladder fibrosis is increasingly recognized as a final common pathological pathway underlying functional deterioration across diverse bladder disorders, with chronic inflammation, extracellular matrix remodeling, and profibrotic signaling contributing to disease progression [12].

BOO, most commonly arising from benign prostatic hyperplasia (BPH), constitutes one of the most prevalent drivers of progressive bladder remodelling in ageing men; contemporary global burden estimates indicate a substantial and growing BPH prevalence with significant associated disability [13]. SCI represents another major upstream aetiology: over 15 million individuals worldwide live with SCI, and bladder dysfunction is among the most clinically conse-quential secondary complications, frequently progressing to a low-compliance, fibrotic bladder wall that threatens upper urinary tract function and long-term survival [14].

Despite meaningful advances in pharmacotherapy, neuromodulation, intravesical botulinum toxin, and recon-structive surgery, a persistent clinical gap remains for therapies capable of reversing established fibrosis, restoring detrusor structure and contractile function, and providing durable remodelling control [2]. MSCs are particularly compelling candidates for bladder regeneration because they can exert broad paracrine antifibrotic, immunomodu-latory, angiogenic, and trophic actions, and under appropriate conditions may adopt bladder-relevant lineage characteristics [15,16]. From a pharmaceutical perspective, engineered cell products delivering specific growth fac-tors—most notably hepatocyte growth factor (HGF), which antagonises the profibrotic actions of transforming growth factor-β1 (TGF-β1)—represent a mechanism-informed “living drug delivery” strategy that aligns with con-temporary expectations for potency-defined, regulatory-grade biological medicines [17,18].

The pharmaceutical focus encompasses cell-based delivery platforms, retention strategies including biomaterial scaffolds and spheroids, mechanistic pathways governing tissue repair, early clinical translation data, and regulatory considerations for advanced therapy medicinal products (ATMPs) in bladder indications.

## 2. Bladder Fibrosis and Neurogenic Dysfunction: Pathophysiology and Therapeutic Rationale

Fibrotic remodelling of the bladder wall is initiated and sustained by a network of interconnected pathological signals, principally involving chronic ischaemia, inflammation-driven activation of resident fibroblasts and myofibroblasts, and augmented TGF-β1/connective tissue growth factor (CTGF)–Smad signalling that promotes collagen deposition while suppressing fibrolytic pathways [1,12]. In BOO, sustained intravesical pressure overload drives smooth muscle hypertrophy, progressive collagen accumulation, and reduced compliance; in SCI-induced neurogenic bladder, loss of supraspinal modulation leads to detrusor overactivity and subsequent hypertrophy, denervation atrophy, and fibrosis that can ultimately produce a small, poorly compliant bladder endangering the upper urinary tract [7].

The HGF/c-Met receptor axis has been extensively characterised as an endogenous counter-regulatory mechanism against TGF-β1-driven fibrogenesis. HGF inhibits profibrotic Smad transcriptional programmes, promoting a shift toward fibrolytic and cytoprotective signalling [19]. MSC-based delivery of HGF therefore provides a pharmacologically rational paracrine therapeutic mechanism for bladder fibrosis, and the engineering of MSC products to overexpress HGF represents an early implementation of “mechanism-defined potency enhancement”—a concept now central to regulatory expectations for advanced cell therapy products [5,17].

## 3. The Six Key Studies from Our Group

The six studies summarised below collectively describe a translational research programme spanning molecular imaging, cell tracking, fibrosis modulation, functional recovery, and engineered cell therapeutics. Importantly, these investigations should be interpreted alongside complementary work from other groups, which has demonstrated similar therapeutic trends using alternative stem-cell sources, delivery routes, and disease models. Differences in efficacy magnitude, durability, and proposed mechanisms likely reflect variations in experimental design and biological context.

### 3.1. Molecular MRI Tracking of hMSCs in Rat and Rabbit Bladders (Song and Ku [3])

This study established the feasibility of non-invasive longitudinal tracking of SPIO-labelled hMSCs in rat and rabbit bladders using clinical 1.5 T MRI hardware [3]. SPIO labelling preserved hMSC viability and tri-lineage (adipogenic/chondrogenic/osteogenic) differentiation capacity; a threshold of ≥1 × 10^5^ labelled cells generated detectable signal hypointensity in vitro; and post-injection T2-weighted MRI demonstrated locally confined signal changes consistent with labelled cell retention for at least 12 weeks, validated by Prussian blue histochemistry.

The principal strength of this work was its demonstration that bladder-directed cell tracking is achievable with clinically deployable MRI hardware, directly anticipating regulatory requirements for biodistribution and retention assessment in cell-based investigational products. A key limitation is that SPIO-based signal reflects iron oxide rather than cell viability per se, since signal can persist after cell death through macrophage-mediated iron uptake [17]. Nonetheless, this paper helped legitimise bladder-targeted cell therapy by offering a practical pharmacokinetic imaging tool for answering the foundational question of where transplanted cells localise and how long they persist.

### 3.2. SPIO-hMSC Therapy for BOO Bladder Fibrosis with MRI Monitoring (Lee et al. [4])

This study constructed a rat BOO model and delivered 1 × 10^6^ SPIO-labelled hMSCs by bladder wall injection two weeks after obstruction induction, followed by serial T2-weighted MRI and cystometric, molecular, and histological fibrosis assessment four weeks post-transplantation [4]. BOO markedly elevated collagen deposition and TGF-β protein expression; hMSC transplantation attenuated these fibrosis markers toward baseline levels. Concomitantly, HGF and c-Met expression increased after MSC delivery, and both maximal voiding pressure and residual urine volume—elevated by BOO—showed functional recovery following transplantation. MRI demonstrated clearly localised hypointense signal at injection sites, providing an imaging proxy for cell retention.

The most distinctive pharmaceutical contribution of this study is the integration of a biological efficacy readout (fibrosis reduction; functional recovery) with molecular imaging confirmation of local cell presence—directly linking the delivery platform, retention, and pharmacodynamic effect within a single experimental framework. Limitations include a relatively short follow-up window (four weeks post-transplantation), the use of young female rats rather than the older male demographic most clinically relevant to BOO, and the inherent interpretive constraints of SPIO-based viability inference [4].

### 3.3. HGF-Overexpressing B10 MSCs for Enhanced BOO Antifibrotic Efficacy (Song et al. [5])

This study introduced engineered MSC potency enhancement as a pharmaceutical strategy for bladder fibrosis [5]. An immortalised human MSC line (BM3.B10) was retrovirally transduced to overexpress HGF (B10.HGF), and bladder wall injection of B10 or B10.HGF cells was performed two weeks after BOO induction. B10.HGF reduced bladder weight, normalised collagen area fraction to near-control levels (approximately 13.3% vs. 26.6% in untreated BOO; controls approximately 12.2%), and restored intercontraction interval. Maximal voiding pressure, elevated after BOO, recovered toward normal after B10.HGF treatment, though residual urine volume was not reversed.

This study represents an early bladder-specific demonstration that overexpression of a defined growth factor can amplify MSC antifibrotic efficacy and normalise tissue collagen burden—a finding that directly foreshadows modern potency assay paradigms and mechanism-informed product design in regenerative medicine [17]. Translational limitations include the use of an immortalised, retrovirally modified cell line, which introduces regulatory concerns regarding tumorigenicity and manufacturing burden, and the incomplete functional recovery of residual urine volume, suggesting that fibrosis attenuation alone may be insufficient when concurrent denervation or persistent outlet obstruction coexists [5].

### 3.4. Bladder Wall Transplantation of B10 MSCs in SCI-Induced Bladder Fibrosis (Lee et al. [6])

This study addressed an underexplored dimension of SCI research—direct bladder organ dysfunction—by delivering fluorescent magnetic nanoparticle-labelled B10 cells into the bladder wall of SCI rats, four weeks after injury [6]. Reduced collagen deposition was observed after B10 transplantation, MR imaging at four weeks confirmed hypointense signal localised to the bladder consistent with labelled cell persistence, and anti-human mitochondria immunostaining corroborated B10 cell survival. Evidence was reported that transplanted cells adopted a smooth muscle cell phenotype. Functionally, intercontraction interval and maximal voiding pressure, both altered by SCI, showed recovery following B10 transplantation.

The conceptual significance of this study lies in its repositioning of SCI bladder research: rather than relying exclusively on spinal cord repair to yield downstream bladder recovery, it demonstrates that direct bladder wall therapy can modulate detrusor remodelling and functional parameters independently. This opens a complementary translational pathway in which combined spinal and bladder-targeted interventions may be systematically evaluated. Limitations include a short follow-up period relative to the chronicity of clinical SCI bladder dysfunction, and the unresolved question of whether apparent smooth muscle differentiation versus paracrine-mediated remodelling was the dominant mechanism of benefit [6].

### 3.5. Systematic Review and Meta-Analysis of Stem Cell Therapy for SCI-Induced Bladder Dysfunction (Kim et al. [7])

This quantitative synthesis reviewed experimental SCI studies published between 1990 and 2012 and meta-analysed urodynamic endpoints including voiding pressure, non-voiding contractions, residual urine volume, and bladder capacity [7]. Eight studies encompassing 224 subjects met numerical inclusion criteria. Stem cell therapy was associated with statistically significant improvements in voiding pressure, non-voiding contractions, and residual urine volume; bladder capacity improvements were noted specifically in transection models. Substantial heterogeneity (high I^2^ for multiple endpoints) was documented.

This study provided early quantitative evidence that preclinical MSC therapy yields partial but measurable urodynamic recovery across SCI models, thereby strengthening translational credibility for bladder-targeted regenerative interventions. Its limitations directly reflect the heterogeneity of its input literature—variable SCI models, diverse cell sources, inconsistent dosing and delivery routes, and probable publication bias—and the work functioned as a rigorous field audit, highlighting the need for harmonised models and standardised urodynamic reporting protocols [7].

### 3.6. HGF-Overexpressing hMSCs for BOO-Induced Underactive Bladder (Kim et al. [8])

This study extended the B10.HGF platform to BOO-induced underactive bladder (UAB), a clinically challenging condition characterised by impaired contractility and incomplete emptying [8]. Transplantation of hMSCs into the bladder wall significantly reversed BOO-induced pathological changes—elevated intercontraction interval, increased residual urine volume, and reduced maximal voiding pressure—and these functional benefits were further amplified in the HGF-overexpressing group. Histologically, hMSC therapy markedly reduced collagen accumulation and suppressed fibrosis-associated gene expression, with more pronounced effects in the HGF-overexpressing cohort. Angiogenesis, assessed by von Willebrand factor-positive microvessel density, was enhanced after hMSC transplantation, and apoptotic activity—elevated in BOO conditions by caspase-3 expression and TUNEL positivity—was significantly reduced, with both effects potentiated by HGF overexpression.

Mechanistically, this study delineated a multifactorial therapeutic model in which hMSCs restore bladder function through (i) TGF-β pathway suppression and fibrosis inhibition, (ii) enhancement of microvascular perfusion, and (iii) reduction in detrusor myocyte apoptosis. The HGF/c-Met signalling axis was identified as a central amplifier of all three regenerative mechanisms. This study is distinctive in directly linking structural remodelling to contractile function restoration—a clinically meaningful endpoint that extends beyond fibrosis attenuation—and in establishing angiogenesis and anti-apoptosis as co-equal therapeutic mechanisms alongside antifibrotic activity [8].

## 4. Thematic Synthesis of the Broader Literature

### 4.1. Cell Sources and Engineering Strategies

Bone marrow-, adipose-, umbilical cord-, and embryonic stem cell-derived MSC lineages dominate bladder regen-eration research owing to their established paracrine secretome breadth and expandability [9]. Recent studies have substantially diversified the cell source landscape. Urine-derived stem cells (USCs) were evalu-ated in a partial BOO rat model, where repeated intravenous USC administration improved bladder compliance, reduced end-filling pressure, and attenuated collagen deposition; miRNA-mRNA expression profiling nominated mechanistic pathways including necroptosis regulation and cytokine networks potentially relevant to bladder repair [10].

Adipose stromal vascular fraction (ad-SVF) spheroids demonstrated superior urodynamic and structural outcomes compared with dispersed SVF in a partial BOO-induced UAB model, with spheroid delivery associated with enhanced angiogenesis, reduced apoptosis, improved cell retention, and higher expression of trophic factors including basic fibroblast growth factor, HGF, and VEGF-A—findings that reinforce the therapeutic relevance of the HGF/angiogenic axis established in the cornerstone programme [20]. Human umbilical cord MSCs have been shown to contribute to bladder function reconstruction after acute SCI through the p38 MAPK/NF-κB signalling pathway [21], and human amniotic fluid stem cells (hAFSCs) reduced bladder weight, wall thickness, collagen-to-smooth muscle ratio, and multiple pro-fibrotic mediators—including TGF-β1 and CTGF—alongside improvements in cystometric parameters in a partial BOO rat model [22]. From a pharmaceutical product perspective, the diversification of cell sources reflects both scientific progress and a regulatory maturation challenge: each source requires distinct product characterisation, potency assay development, and clinical risk assessment, underscoring the need for condition-specific translational assumptions rather than a generic “MSC therapy” framework [9].

Therapeutic efficacy may vary according to stem-cell source because distinct MSC populations exhibit differences in secretome composition, proliferative capacity, immunomodulatory activity, and angiogenic potential. Consequently, direct comparison between studies remains challenging. Clinical translation increasingly requires source-specific characterization, standardized manufacturing procedures, and mechanism-linked potency assays to ensure reproducibility and product consistency.

### 4.2. Delivery Routes, Biomaterial Platforms, and Retention Strategies

A persistent barrier to effective bladder-directed cell therapy is achieving sufficient wall exposure and therapeutic retention, particularly for minimally invasive approaches where urothelial barriers, urine washout, and mucus layers impede residence time [23]. The cornerstone studies employed direct bladder wall injection to maximise local delivery, providing a biological efficacy benchmark that alternative platforms aim to reproduce or exceed with less invasive administration.

Spheroid and cell-aggregate formats have emerged as a promising pharmaceutical strategy to enhance both retention and paracrine potency. The ad-SVF spheroid study demonstrated imaging-confirmed retention advantages over dispersed cell preparations, attributing improved outcomes to three-dimensional cell organisation that amplifies secretory activity [20]. Adipose-derived MSC cell sheets, investigated in a SCI-related neurogenic bladder context, were shown to promote axonal regeneration and restore voiding recovery in SCI rats, positioning cell sheet transplantation as a scalable delivery alternative for defined tissue surfaces [24]. Hydrogel-based delivery systems, while predominantly explored in intravesical drug delivery, provide a directly applicable engineering framework for extending the residence time of cell or extracellular vesicle-based therapeutics within the bladder lumen or wall [25]. A recent advance employing magnetically controlled microgelbots loaded with stem cells demonstrated targeted delivery and retention within the bladder, offering a novel platform for treating interstitial cystitis with spatial precision [23].

### 4.3. Mechanisms of Action

Across bladder conditions studied, the following convergent mechanisms can be integrated into a coherent pharmacological model: Antifibrotic pathway modulation via downregulation of TGF-β1/CTGF/Smad signalling and reduction in collagen deposition represents the dominant mechanistic theme across both cornerstone and complementary BOO/SCI studies [4,22]. HGF antagonises TGF-β1 profibrotic activity by stabilising the Smad transcriptional co-repressor TGIF, thereby suppressing downstream collagen synthesis programmes [19]. The cornerstone BOO studies established that endogenous HGF and c-Met upregulation accompanies MSC therapy, and that exogenous HGF overexpression substantially amplifies antifibrotic outcomes—positioning this axis as a validated, mechanism-informed pharmaceutical target for cell product engineering [5,8,17,18].

**Figure 1:**
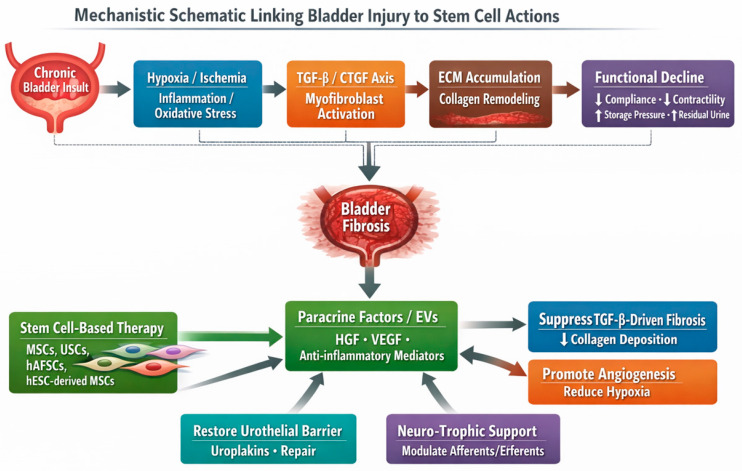
Mechanistic schematic linking bladder injury to stem cell actions. Colors of the boxes indicate different functional categories: red, initial bladder insult; blue, pathophysiological stress responses; orange, cellular and mo-lecular activation pathways; brown, extracellular matrix (ECM) remodeling; purple, functional consequences of fibrosis; green, stem cell-based therapeutic interventions and paracrine mediators; teal and violet, tissue repair and neurotrophic supportive effects. Solid arrows indicate progression or promotion of biological processes, whereas dashed lines represent feedback interactions and indirect relationships that may contribute to the development and perpetuation of bladder fibrosis. Abbreviations: TGF-β, transforming growth factor-β; CTGF, connective tissue growth factor; ECM, extracellular matrix; EVs, extracellular vesicles; HGF, hepatocyte growth factor; VEGF, vascular endothelial growth factor; MSCs, mesenchymal stem cells; USCs, urine-derived stem cells; hAFSCs, human amniotic fluid-derived stem cells; hESC-derived MSCs, human embryonic stem cell-derived mesenchymal stem cells.

Anti-inflammatory and immunomodulatory effects are consistently reported in BOO and interstitial cystitis models, with reductions in inflammatory cytokine expression, macrophage infiltration, and immune cell activation markers following MSC administration [22]. Angiogenesis and anti-apoptosis have been mechanistically linked to bladder recovery in the UAB model, where HGF-overexpressing hMSC therapy augmented microvessel density and reduced detrusor myocyte apoptosis—both of which represent tractable pharmacodynamic biomarkers for future potency assay development [8,20]. Neuro-urothelial restoration has been demonstrated in radiation cystitis, where MSC therapy preserved urothelial integrity, reduced vascular damage, and maintained uroplakin expression, extending the therapeutic rationale beyond fibrosis attenuation to encompass epithelial barrier restoration [26]. The major mechanisms linking bladder injury, fibrosis progression, and the therapeutic actions of stem cell–based interventions are summarized in Figure 1.

## 5. Animal Models and Functional Endpoints

The cornerstone studies employed clinically interpretable models—BOO-induced fibrosis and cystometric impairment, and SCI-induced neurogenic bladder dysfunction with fibrosis—using reproducible rat model paradigms [4,7]. The broader literature emphasises the importance of model selection (partial vs. complete obstruction; SCI contusion vs. transection; timing of intervention relative to disease establishment) and underscores that BOO, SCI, and diabetic cystopathy generate distinct remodelling signatures with different collagen composition, smooth muscle phenotype, and neurochemical profiles, reinforcing the need for condition-specific translational validation rather than cross-indication extrapolation [27].

A consensus emerging since the 2015 meta-analysis [7] is that functional urodynamic endpoints must be paired with mechanistic tissue biomarkers (collagen quantification, TGF-β/CTGF pathway markers, apoptosis indices, microvessel density) and clinically relevant follow-up durations. Compliance and storage pressure thresholds relevant to upper urinary tract protection represent particularly important endpoints that should be explicitly incorporated into future model harmonisation efforts.

## 6. Clinical Translation: Current Evidence and Emerging Trials

The clinical landscape has matured substantially over the past five years across several bladder indications. A first-in-human phase I experience of transurethral submucosal injection of hESC-derived MSCs in three Interstitial cystitis/bladder pain syndrome (IC/BPS) patients reported safety and preliminary efficacy signals, with the authors explicitly noting that dose optimisation remains necessary [11]. A subsequent single-centre, randomised, double-blind phase I/IIa trial (MR-MC-01; hESC-derived MSC therapy) enrolled 22 patients and reported no serious drug-related adverse events alongside improvements in symptom scores and Hunner lesion resolution at six months, with ongoing longer-term follow-up [28]. These clinical findings conceptually align with the cornerstone programme’s emphasis on local bladder wall delivery as the primary route for achieving intramural tissue remodelling.

A 2023 open clinical trial of autologous adipose-derived MSCs in nine men with DU used cystourethroscopy-guided intravesical injection at five bladder wall sites, reporting significant improvements in uroflow metrics and post-void residual volume, with most participants reducing or discontinuing catheterisation over six months and no complications observed [29]. This directly translates the BOO/UAB preclinical programme [8] to a clinical setting and supports the pharmaceutical feasibility of locally delivered autologous cell products for contractile restoration. Two phase I/II trials employing autologous adipose-derived MSCs delivered intraurethrally for male post-prostatectomy incontinence and female stress urinary incontinence reported procedural feasibility and absence of adverse effects, with objective pad-test improvements in subsets of participants, underscoring safety while highlighting the need for controlled efficacy confirmation [30].

A randomised, open-label phase II trial demonstrated that combined intrathecal autologous bone marrow MSC and Schwann cell therapy improved urodynamic parameters and incontinence-related quality of life in complete SCI patients [31]. This neuraxial delivery approach is complementary rather than contradictory to the cornerstone “direct bladder wall repair” paradigm, suggesting that integrated spinal and bladder-targeted strategies may ultimately provide superior outcomes compared with either modality alone [6].

Although the overall safety profile of stem cell-based interventions has been favorable in both preclinical and early clinical studies, potential risks remain important translational considerations. These include unwanted differentiation, ectopic tissue formation, immunogenic reactions, thromboembolic events following systemic administration, and theoretical tumorigenicity associated with genetically modified or extensively expanded cell products. Furthermore, safety profiles may vary according to stem-cell source. Bone marrow-, adipose-, umbilical cord-, and embryonic stem cell-derived products differ in proliferative capacity, immunogenicity, genomic stability during ex vivo expansion, and manufacturing complexity. Additional concerns include donor-to-donor variability, potential immune sensitization following repeated administration, and genetic or epigenetic alterations acquired during prolonged culture. Long-term post-treatment surveillance will therefore be essential to establish the safety profile of bladder-directed cell therapies.

## 7. Regulatory Landscape for Cell-Based Bladder Therapies

The cornerstone programme’s use of SPIO/MNP labelling to localise transplanted cells in vivo remains methodologically relevant to regulatory biodistribution assessment, though modern interpretive standards emphasise the distinction between iron signal and cell viability and advocate for complementary approaches such as clinically approved ferumoxytol-based MRI or bioluminescence reporter systems [16,17]. The FDA provides specific pathways for human cell and tissue-based products, including minimal manipulation/homologous use criteria and expedited Regenerative Medicine Advanced Therapy (RMAT) designation [15]. The December 2024 FDA approval of remestemcel-L-rknd (Ryoncil) for paediatric steroid-refractory acute graft-versus-host disease represents the first FDA-approved MSC therapy and signals meaningful regulatory maturation for MSC product evaluation—a precedent with relevance for future bladder indications pursuing regulated development pathways [32].

In Europe, the European Medicines Agency (EMA) oversees ATMPs under Regulation (EC) No 1394/2007, encompassing both cell-based medicinal products and tissue-engineered products [33]. The trajectory of darvadstrocel (Alofisel)—an adipose stem cell product for complex perianal fistula conditionally authorised in the EU in 2018 and withdrawn in December 2024 due to inability to provide sufficient confirmatory effectiveness data post-authorisation [34]—carries an important translational lesson: conditional approval creates continuing evidence obligations that, if unmet, lead to market withdrawal. Cell therapy developers in bladder indications should plan prospectively for robust post-authorisation confirmatory study designs. Scientific and ethical standards. The International Society for Stem Cell Research guidelines [35] set expectations for rigorous preclinical evidence, transparent clinical reporting, and opposition to premature commercialisation—standards that are fully aligned with the evidence quality and translational rigour demanded by regulatory authorities.

### Regulatory Gaps in the Six Key Studies

While the six key studies generated important preclinical evidence supporting stem cell-based bladder regeneration, several aspects expected under contemporary regulatory frameworks were incompletely addressed. Molecular MRI studies provided valuable biodistribution information but did not directly assess cell viability. The HGF-overexpressing MSC studies demonstrated enhanced biological activity but lacked formal potency assays, long-term tumorigenicity evaluation, and comprehensive product comparability assessments. Furthermore, standardized release criteria and mechanism-based potency testing were not incorporated. These limitations largely reflect the regulatory expectations at the time the studies were conducted and highlight critical areas for future translational development.

## 8. Comparative Summary of Cornerstone and Complementary Studies

A comparative overview of the six key studies and representative complementary studies is presented in Table 1. The translational characteristics of the six key studies, including treatment timing and follow-up duration, are summarized in Table 2.

## 9. Persistent Gaps and Future Directions

### 9.1. Durability and Long-Term Safety

The majority of preclinical bladder studies, including the cornerstone BOO and SCI intervention studies, employ follow-up windows of four weeks post-transplantation—a duration that is insufficient to resolve questions about fibrosis relapse, long-term functional maintenance, late adverse events, or sustained engraftment [4]. Clinical durability data from ongoing IC/BPS and DU trials with multi-year follow-up planning will be essential to establish the therapeutic viability of cell-based bladder regeneration. Importantly, none of the intervention studies formally assessed fibrosis relapse following withdrawal of therapy or included extended treatment-free observation periods, limiting conclusions regarding long-term durability.

### 9.2. Cell Product Standardisation and Recent Advances in Potency Assay Development

Cross-study heterogeneity in cell source, manufacturing protocol, potency characterisation, and delivery route makes comparative analysis methodologically difficult and complicates regulatory translation [9]. The HGF-engineering strategy established in the cornerstone BOO studies [5,8] provides a mechanistically grounded prototype for potency-defined product design, but modern regulatory agencies expect quantitative potency assays demonstrating a measurable pharmacodynamic endpoint—such as antifibrotic activity in co-culture systems, immunomodulatory indices, or angiogenic capacity—that correlates with in vivo therapeutic outcome. Developing such assays specifically for bladder-directed cell products represents a high-priority translational objective.

Recent advances in potency assay development have shifted from simple phenotypic characterization toward mechanism-linked functional testing. Examples include collagen suppression assays in fibroblast co-culture systems, macrophage polarization assays for immunomodulatory activity, endothelial tube formation assays for angiogenic potential, and quantitative HGF secretion measurements for engineered MSC products. Such assays may improve batch-to-batch consistency and facilitate regulatory approval of bladder-directed cell therapies.

### 9.3. Outcome Standardisation and Model Harmonisation

The heterogeneity documented in the 2015 meta-analysis [7] underscores the need for standardised urodynamic protocols, validated bladder histomorphometry methods, and consensus on clinically meaningful endpoints—particularly storage compliance thresholds relevant to upper urinary tract protection and functionally defined contractility recovery criteria. Comparative model studies explicitly evaluating BOO, SCI, and diabetic cystopathy within unified experimental frameworks will facilitate condition-specific translational design assumptions [27].

### 9.4. Imaging-Pharmacokinetic Integration

Advanced imaging modalities—including clinically approved iron-based agents such as ferumoxytol, bioluminescence reporter systems, and 19F MRI—should be integrated as pharmacokinetic readouts in future preclinical and early clinical studies to provide rigorous, quantitative evidence for cell biodistribution, retention kinetics, and persistence duration [16,17]. Linking imaging-based pharmacokinetic data to pharmacodynamic biomarker trajectories would substantially strengthen the regulatory evidence package for bladder-directed cell therapies.

### 9.5. Sex-Specific Considerations

Several preclinical studies discussed in this review employed young female rodents, particularly in BOO models, because of technical considerations and reduced perioperative morbidity. However, bladder outlet obstruction predominantly affects ageing male patients in clinical practice. Biological sex may influence inflammatory responses, fibrosis progression, hormonal regulation, and MSC-mediated tissue repair. At present, evidence regarding sex-specific differences in therapeutic efficacy remains limited. Future investigations should incorporate aged male models and sex-stratified analyses to improve translational relevance and facilitate clinical extrapolation.

## 10. Conclusions

The six key studies from our group contribute to a coherent translational framework linking molecular imaging, antifibrotic therapy, engineered cell products, and functional bladder recovery: establishing MRI-based cell tracking feasibility in the bladder, demonstrating local MSC antifibrotic efficacy in BOO and SCI, introducing HGF-enhanced MSC potency as a mechanism-informed pharmaceutical strategy, and providing quantitative meta-analytic support for partial urodynamic recovery. The distinctive contribution of this programme—integrating local bladder wall delivery, molecular imaging pharmacokinetics, and growth factor-based potency engineering—closely aligns with contemporary regulatory and scientific expectations for advanced cell-based medicines.

The broader literature converges on the same antifibrotic, angiogenic, and immunomodulatory themes, with emerging evidence supporting multiple complementary cell sources and retention-enhancing delivery platforms. The most clinically advanced translation signals are in IC/BPS and detrusor underactivity, where locally delivered cell products have demonstrated safety and encouraging early efficacy in small clinical cohorts. The December 2024 FDA approval of the first MSC therapy and the cautionary withdrawal of darvadstrocel from the EU market collectively underscore that rigorous mechanism-informed product development, robust confirmatory clinical evidence, and post-authorisation compliance are prerequisites for durable regulatory success in the bladder regeneration field.

Importantly, these studies should be viewed as part of a broader international effort to develop regenerative therapies for bladder dysfunction. Continued progress will require rigorous potency assay development, long-term safety assessment, standardized manufacturing procedures, and regulatory alignment to enable successful clinical translation.

## Figures and Tables

**Table 1:** Comparative summary of cornerstone studies and key complementary evidence in stem cell-based bladder therapy.

Study (Year)	Model/Population	Cell Product	Delivery	Primary Outcomes	Follow-Up	Mechanism of Action	Key Findings	Main Limitations
Song & Ku, 2007 [3]	Rat & rabbit bladder (tracking only)	SPIO-labelled hMSCs	Bladder wall injection	MRI detectability; Prussian blue histology; viability/differentiation	≥12 weeks	Biodistribution and cell tracking	MRI visualisation of labelled cells for ≥12 weeks; viability preserved after SPIO labelling	SPIO signal ≠ viability; no disease model
Lee et al., 2012 [4]	Rat BOO	SPIO-labelled primary hMSCs	Bladder wall injection	Collagen/TGF-β; HGF/c-Met; cystometry; MRI	4 weeks post-transplant	Antifibrotic activity through TGF-β suppression and HGF/c-Met activation	Reduced fibrosis markers; improved cystometry; MRI-confirmed local signal	Short follow-up; female rats; SPIO limitations
Song et al., 2012 [5]	Rat BOO	B10 vs. B10.HGF immortalised hMSCs	Bladder wall injection	Bladder weight; collagen area; cystometry	4 weeks post-transplant	HGF-enhanced antifibrotic signaling	B10.HGF normalised collagen area; ICI recovered; residual urine not reversed	Immortalised + gene-modified product; partial functional recovery
Lee et al., 2015 [6]	Rat SCI → bladder fibrosis	MNP-labelled B10 hMSCs	Bladder wall injection at 4 weeks post-SCI	Collagen; cystometry; MRI; anti-human mitochondria IHC	4 weeks post-transplant	Antifibrotic remodeling and smooth muscle restoration	Reduced fibrosis; MRI signal at 4 weeks; ICI and MVP recovered; smooth muscle differentiation reported	Durability unknown; broader urinary outcomes not assessed
Kim et al., 2015 [7]	SCI (meta-analysis; 8 studies; n = 224)	Various stem cell types	Various routes	Voiding pressure; NVC; residual urine; bladder capacity	Variable across studies	Quantitative evaluation of functional recovery	Significant improvements in voiding pressure, NVC, and residual urine; high heterogeneity	Variable study quality; model heterogeneity; likely publication bias
Kim et al., 2021 [8]	Rat BOO → UAB (5 groups; n ≈ 50)	hMSC vs. HGF-overexpressing hMSC	Bladder wall injection	Cystometry; collagen/TGF-β; angiogenesis (vWF); apoptosis (caspase-3/TUNEL)	4 weeks post-transplant	Antifibrotic, angiogenic, and anti-apoptotic effects	HGF-hMSC restored contractility; reduced fibrosis, apoptosis; enhanced angiogenesis	Immortalised + gene-modified product; partial recovery of residual urine
Tu et al., 2022 [10]	Rat partial BOO	Human USCs (repeated IV)	Intravenous (biweekly)	Compliance; MVP; collagen; miRNA-mRNA profiling	Longitudinal (7–19 weeks)	Transcriptomic regulation	Improved bladder function and remodelling; mechanistic pathway hypotheses via transcriptomics	IV route retention limitations; single centre
Liu et al., 2022 [20]	Rat partial BOO → UAB	ad-SVF spheroids	Bladder tissue administration	Urodynamics; angiogenesis; apoptosis; growth factor expression	4 weeks	Enhanced retention and trophic factor secretion	Spheroids improved function and retention; elevated HGF/VEGF-A supports trophic mechanism	SVF product heterogeneity; translation requires definition
Liang et al., 2022 [22]	Rat partial BOO	Human amniotic fluid stem cells	Post-BOO treatment	TGF-β1/CTGF; inflammatory markers; cystometry	2–6 weeks	TGF-β1/CTGF inhibition	Improved detrusor dysfunction; reduced inflammatory and profibrotic markers	Mechanism mostly associative; dose optimisation required
Brossard et al., 2023 [26]	Chronic radiation cystitis (rat)	Adipose-derived MSCs	Intravenous	Haematuria; urothelial/vascular damage; uroplakin expression	3–12 months	Urothelial and vascular repair	Reduced vascular/urothelial damage; barrier restoration mechanism	Human dose/manufacturing translation needed
Shin et al., 2022 [11]	IC/BPS (Hunner-type), humans; n = 3	hESC-derived MSCs	Transurethral/submucosal injection	Safety; symptom signals	Short-term	Clinical feasibility and safety	First human IC application; safe, feasible; potential efficacy	Very small cohort; dose refinement required
Kyung et al., 2025 [28]	IC (Hunner lesions), humans; n = 22	MR-MC-01 (hESC-derived MSC)	Bladder submucosal injection	Safety; symptom scores; lesion resolution	6 months (ongoing)	Clinical efficacy and lesion resolution	No serious drug-related AEs; symptom and lesion improvements	Larger trials, durability, and reproducibility needed
Coelho et al., 2023 [29]	Detrusor underactivity, humans; n = 9	Autologous ADSCs	Intravesical injection (5 points)	Uroflow; voided volume; post-void residual; QoL	6 months	Functional contractility restoration	Significant improvements; many stopped catheterisation; no complications	Small uncontrolled cohort; patient selection essential

**Table 2:** Translational Characteristics of the Six Key Studies.

Study	Disease Model	Treatment Timing	Follow-Up	Fibrosis Relapse Assessed	Treatment-Free Observation
Song & Ku [3]	Normal bladder	Immediate	12 weeks	No	Yes
Lee et al. [4]	BOO	2 weeks after BOO	4 weeks	No	No
Song et al. [5]	BOO	2 weeks after BOO	4 weeks	No	No
Lee et al. [6]	SCI	4 weeks after SCI	4 weeks	No	No
Kim et al. [7]	Meta-analysis	Variable	Variable	Variable	Variable
Kim et al. [8]	BOO-induced UAB	2 weeks after BOO	4 weeks	No	No

ADSC, adipose-derived stem cell; AE, adverse event; BOO, bladder outlet obstruction; IC/BPS, interstitial cystitis/bladder pain syndrome; ICI, intercontraction interval; hESC, human embryonic stem cell; hMSC, human mesenchymal stem cell; MNP, magnetic nanoparticle; MVP, maximal voiding pressure; NVC, non-voiding contractions; QoL, quality of life; SCI, spinal cord injury; SPIO, superparamagnetic iron oxide; TGF-β, transforming growth factor-β; UAB, underactive bladder; USC, urine-derived stem cell; vWF, von Willebrand factor.

## Data Availability

No new data were created or analyzed in this study. Data sharing is not applicable to this article.
